# Generating a Mediation Model of Moral Cost and Aggression

**DOI:** 10.3390/bs16030463

**Published:** 2026-03-20

**Authors:** Jing Lin, Yang Hu, Jia-Ming Wei, Ling-Xiang Xia

**Affiliations:** 1 Research Center of Psychology and Social Development, Faculty of Psychology, Southwest University, Chongqing 400715, China; linjinglj@email.swu.edu.cn; 2 Key Laboratory of Cognition and Personality (Southwest University), Ministry of Education, Chongqing 400715, China; 3 School of Intelligent Engineering, Chongqing City Management College, Chongqing 401331, China; huyang@cqc.edu.cn; 4 College of Education, Huaibei Normal University, Huaibei 235000, China; weijm@chnu.edu.cn

**Keywords:** aggression, moral cost, positive outcome expectancies for aggression, subjective value, protective factors

## Abstract

The effects of moral protective factors (e.g., moral cost) on aggression and the underlying mechanisms remain unclear. To address this issue, this study developed the Moral Cost of Aggression Questionnaire (MCAQ) and validated its psychometric properties in 516 college students (287 female; *M*age = 19.77 years, *SD* = 1.61). Subsequently, the relationships among moral cost, positive outcome expectancies for aggression (POEA), and aggression were examined in 749 college students (330 females; *M*age = 18.96 years, *SD* = 0.74). Mediation analysis indicated that POEA mediated the relationship between moral cost and aggression. This pattern of associations is consistent with the hypothesis that moral cost is negatively associated with aggression, in part through its link to lower subjective value of aggressive outcomes (i.e., lower POEA). This study provides a reliable and valid measure of the trait moral cost (MCAQ) and offers preliminary empirical support for a discounting mechanism in which moral cost is associated with reduced aggression via decreased POEA. These findings suggest that interventions targeting both moral cost and outcome valuation may be a useful direction for future research.

## 1. Introduction

Aggression refers to a response or tendency towards harmful behavior with the intention of causing harm; such harm is something the other party wants to avoid (Anderson & Bushman, 2002; R. Li & Xia, 2021). Aggression can harm both victims and perpetrators among college students (Fite et al., 2012; Salmivalli & Nieminen, 2002; Xu & Zhang, 2008). Therefore, identifying factors that may increase or decrease aggression is important when designing targeted prevention and intervention strategies. Nonetheless, the majority of current research (Jakubovic & Drabick, 2020; W. Li et al., 2022; Uçur & Dönmez, 2023) has primarily focused on risk factors that increase aggression while largely neglecting protective factors, especially moral protective factors (e.g., moral cost). Recent studies (Dhillon et al., 2023; Keum & Meier, 2024) have demonstrated that moral cost functions as a significant protective factor against immoral behavior, indicating its potential as a crucial moral protective factor against aggression. Nevertheless, its effect on aggression and the underlying mechanisms remain unclear. To fill this gap, this research will first develop a Moral Cost of Aggression Questionnaire (MCAQ) to measure moral cost. Subsequently, utilizing this newly developed instrument, the study will primarily examine the relationship between moral cost and aggression, and its potential mediating mechanism (e.g., positive outcome expectancies for aggression, POEA). Elucidating this mechanism will present more targeted prevention strategies for aggression. This study focuses on the relationship between moral cost and aggression among college students because this period represents a critical period in the transition from late adolescence to early adulthood, during which moral cognition and values are still developing (X. Li et al., 2024; Lin et al., 2026). And it is thus a key time for intervening in aggression. Second, college students face multiple stressors and frustrations, which may serve as triggers for aggression (Malaviya, 2025; Thomas, 2019). Therefore, investigating moral protective factors (e.g., moral cost) against aggression and their underlying mechanisms among college students is of significant importance.

Moral cost is an intrinsic cost (Dhillon et al., 2023) comprising moral cognition and emotions that serve as self-punishment (R. Nelissen & Zeelenberg, 2009; R. M. Nelissen, 2012; Zimmerman, 2008), associated with both the act itself and its expected outcomes. One important moral cost is the negative moral emotions that one anticipates experiencing when engaging in aggressive behavior (van Gelder et al., 2022), encompassing harm aversion, shame, and guilt (Bachman et al., 1992; Grasmick & Bursik, 1990; Grasmick et al., 1993; Nagin & Paternoster, 1993; Qu et al., 2022). It is crucial to distinguish between trait moral cost and state moral cost. Trait moral cost refers to an individual’s stable and enduring disposition to perceive moral discomfort. It somewhat reflects a baseline sensitivity to moral norms and represents a relatively invariant personality trait. In contrast, state moral cost refers to the immediate moral emotional experience generated by an individual in a specific context, which can be dynamically influenced by factors such as moral disengagement or neutralization techniques (Bandura et al., 1996; Sykes & Matza, 2017). The present study focuses on trait moral cost. We posit that trait moral cost constitutes a fundamental protective factor against aggression. While state factors can temporarily attenuate moral perception, an individual with high trait moral cost possesses a stronger initial inhibitory threshold. Therefore, this study focuses on the relationship between trait moral cost and aggression, as well as the underlying mechanisms.

However, there are some problems with the existing instruments for measuring the moral cost. First, they usually only assess a subset of the components (e.g., use moral sensitivity as an indicator of moral cost), which means they fail to provide a comprehensive assessment (Barile et al., 2025; Rosas et al., 2023). Secondly, these tools usually use general depictions about aggression instead of focusing on specific aggressive behaviors (e.g., would you feel guilty later if you were to use violence?) (van Gelder et al., 2022). To investigate the inhibitory effect of moral cost on aggression and its underlying mechanisms, this study will develop a measurement tool that focuses on specific aggressive behaviors and integrates the assessment of various fundamental components of trait moral cost, thereby addressing the limitations of existing measures. While moral cost encompasses multiple components (such as moral cognition, various moral emotions), individuals need to integrate diverse moral emotions into a holistic cost based on their moral cognition when engaging in aggression. Therefore, it can be hypothesized that moral cost is a unidimensional construct that captures this integrated perception. Thus, this study assumes that moral cost is unidimensional.

The function of moral cost is inhibiting immoral behavior (Dhillon et al., 2023; Keum & Meier, 2024). A quasi-experimental study demonstrated that moral cost can inhibit immoral behaviors, such as wrongful dismissal (Keum & Meier, 2024). Furthermore, research in the field of aggression (Cen et al., 2025; Crockett et al., 2010, 2015; Martinez et al., 2024; Perera et al., 2016) has identified that components of moral cost—such as harm aversion, guilt, and empathy—serve to inhibit aggression. This evidence collectively indicates that moral cost serves as a significant protective factor against aggression.

We posit that moral cost has an inhibitory effect on aggression for two primary reasons. The first is moral self-punishment. The immediate sources and components of moral costs are moral self-sanctions (e.g., moral self-condemning, feelings of guilt). When a person’s behaviors go against moral and social norms, these emotions and cognitions of self-punishment are activated, which inhibits aggression (Dhillon et al., 2023; Levitt & List, 2007). In summary, the self-punishing function of moral cost directly inhibits aggression (Kaplow & Shavell, 2007; R. Nelissen & Zeelenberg, 2009; van Gelder et al., 2022; Zimmerman, 2008). The second is that moral cost can reduce the subjective value of aggressive outcomes, leading to a decrease in aggression. Given that POEA inherently involves an evaluation for the subjective value of aggressive outcomes (Allen et al., 2018; Lin & Xia, 2026; Wei & Xia, 2023), it follows that moral cost may inhibit aggression by decreasing POEA. The specific rationale for this pathway is elaborated below.

POEA refers to the subjective estimation of the value of possible and favorable outcomes from harming (Allen et al., 2018; Lin & Xia, 2026; Wei & Xia, 2023), and this evaluation is not fixed but malleable (Mischel, 1973). Thus, it is used to represent the subjective value of aggressive outcomes. POEA originates from social learning theory and social cognitive theory (Bandura, 1973; Crick & Dodge, 1994, 1996) and is considered a core cognitive motivation driving aggression, particularly instrumental or proactive aggression. In this study, POEA serves as a critical cognitive variable linking moral cost to aggression, for the following reasons: Firstly, moral cost, as a discount factor, can result in a decrease in subjective value (manifests as decreasing POEA) by discounting objective value (e.g., aggression reward). Specifically, a core function of any cost is to discount the objective value of a commodity, thereby leading to a decrease in subjective value (Białaszek et al., 2019; Escobar et al., 2023; Mitchell, 2003, 2017). In the context of aggression, we posit that moral cost functions as a discount factor. The objective value of aggression refers to the rewards gained from engaging in it, such as obtaining resources or establishing dominance. However, when an individual anticipates the moral cost (e.g., guilt, shame) associated with aggression, this cost discounts the objective value of the aggressive outcomes. The result of this discounting process is a lowered subjective value of those outcomes.

To further clarify the causal relationship between moral cost and POEA, we draw on the fundamental principle of subjective value formation as revealed by the classic discount function (Cosenza et al., 2017; Escobar et al., 2023; Jones & Rachlin, 2006) V = A/(1 + kD): the subjective value (V) of an option is formed after its objective value (A) is discounted by a cost (D). Applying this model to the context of aggression, the reward of aggression constitutes its objective value (A), while the moral costs induced by aggression can be regarded as the cost (D). Moral cost leads to a decrease in the subjective value (V)—that is, a reduction in POEA—by discounting the objective value of aggression (A). Therefore, from the perspective of the discount function, moral cost is a factor that precedes and determines the formation of subjective value (e.g., POEA). A recent study (Barile et al., 2025) provides evidence for this point. By integrating moral cost into an individual decision-making framework, they found that as moral cost increases, the overall utility decreases (essentially, it is the subjective value of behavioral outcomes), thereby reducing their willingness to engage in unethical behaviors like tax evasion or welfare fraud.

Secondly, empirical research has consistently demonstrated that POEA serves as a significant positive predictor of aggression (Chen & Sun, 2025; Crick & Dodge, 1996; Lin & Xia, 2026). For example, a study using a reward interference task found that participants with high POEA were found to exhibit higher levels of proactive aggression (Chen & Sun, 2025); a recent longitudinal study indicated that POEA can predict aggression 6 months later (Lin & Xia, 2026). POEA represents the goals and reasons for aggression, reflecting the cognitive motivation of wanting to engage in it (Crick & Dodge, 1994; Richetin et al., 2011). Thus, it is possible that POEA predicts aggression.

Finally, prior studies (Cano et al., 2014; Liao et al., 2020) have demonstrated that positive outcome expectancies can mediate the relationship between negative emotions and behaviors (e.g., smoking). We propose that POEA serves as a mediator in the relationship between moral cost and aggression. Specifically, moral cost reduces the subjective value of aggression outcomes (i.e., decreases the level of POEA), which diminishes the motivation for aggression and ultimately leads to a reduction in aggression.

This study aims to elucidate moral cost as a novel protective factor against aggression and to investigate its underlying mechanism among college students. To this end, the research will first develop a trait moral cost questionnaire. Subsequently, we propose a mediation model to examine the relationships between moral cost, POEA, and aggression. Specifically, moral cost not only directly and negatively predicts aggression but also exerts an indirect effect on it by reducing POEA.

Study 1 aimed to develop a questionnaire suitable for measuring trait moral cost in the context of specific aggressive behaviors. We first administered the MCAQ to a college student sample (Sample 1) to test its psychometric properties. Study 2 aimed to test the proposed mediation model. Moral cost, POEA, and aggression were assessed in another sample (Sample 2). The collected data were subsequently analyzed to evaluate the proposed mediation model.

## 2. Study 1

### 2.1. Participants

Based on recommendations for factor analysis, which requires a minimum sample size of 200 or at least five times the number of questionnaire items (Kotrlik & Higgins, 2001), and considering the planned initial item pool of about 10 items, we set a target sample size of 500 participants to ensure sufficient data for factor analysis. College students were recruited from three universities in two locations in China via a convenience sampling method. With the assistance of psychology instructors, an online questionnaire was distributed to 544 students during class intervals. Participants first read the informed consent page, participated voluntarily, and received minor course credit upon completing the questionnaire. The data were subsequently screened based on several criteria: response consistency, attention check items, and validity examination items. Following this screening process, 516 valid questionnaires were retained (287 female; *M*age = 19.77 years, *SD* = 1.61), yielding an effective response rate of 94.85%. The research protocol was approved by the Institutional Review Board of Faculty of Psychology at our institution (H21056).

### 2.2. Measures

To develop the initial version of the MCAQ, we first examined the literature on moral cost to delineate its core components (e.g., harm aversion, guilt, shame, and empathy for the victim) (Abbink & Herrmann, 2011; Attanasi et al., 2019; Euler et al., 2017; Qu et al., 2022; Rothenhäusler et al., 2015; van Gelder et al., 2022). Based on this, we operationalized moral cost as the degree to which individuals anticipate experiencing these integrative negative emotions when engaging in aggressive behavior. The initial MCAQ measured the moral cost associated with ten specific aggressive behaviors. To ensure the relevance of the assessed behaviors, we adopted the list of these ten behaviors from the Aggressive Outcomes Expectancy Questionnaire (AOEQ; Wei & Xia, 2023). Which ensured that the MCAQ and AOEQ referenced the same behavioral contexts. Subsequently, we consulted a psychology professor and a few graduate students on the clarity, content relevance, and face validity of the items, on the basis of which the initial version of the MCAQ was formed. Crucially, all item content of the MCAQ was newly developed to uniquely assess the moral cost of each behavior. For each of the 10 aggressive behaviors (e.g., arguing or quarreling, hitting someone), participants were asked to rate the extent to which they would generally feel discomfort, guilt, and shame when engaging in the behavior, as well as the extent to which they would experience the pain and distress felt by the victim. Referencing the AOEQ (Wei & Xia, 2023) and the Cognitive Appraisal of Risky Events Questionnaire (Fromme et al., 1997), responses were recorded on a 7-point Likert scale ranging from 1 (extremely low) to 7 (extremely high). A higher mean score on this questionnaire reflects a higher moral cost. The initial version of the MCAQ consisted of 10 items, with a complete list provided in Table 1.

### 2.3. Statistical Analysis

SPSS 26 and Mplus 8.3 were used to analyze the data. The analytical procedure was as follows. First, item analysis was conducted. The item-total correlation and the critical ratio (CR) were used to assess the discrimination of each item, with items flagged for removal if they failed to meet preset criteria (item-total correlation < 0.30; non-significant CR). Second, Exploratory Factor Analysis (EFA) was performed. The suitability of the data for EFA was assessed using the Kaiser–Meyer–Olkin (KMO) measure and Bartlett’s test of sphericity. Items were removed based on preset criteria (e.g., communality < 0.30, factor loading < 0.40, a factor constituted with fewer than three items). Third, reliability analysis was conducted. Cronbach’s alpha coefficient was used to check the internal consistency reliability. Fourth, Confirmatory Factor Analysis (CFA) was performed on the retained items to test the hypothesized structure. Furthermore, criterion validity was assessed through the MCAQ’s total score associations with established validation measures.

### 2.4. Results

#### 2.4.1. Item Analysis

The results indicated that scores for each item were significantly and positively correlated with the MCAQ’s total score. Furthermore, independent-samples *t*-tests revealed significant differences in the scores for each item between the high-score group (MCAQ’s total score is in the top 27%) and the low-score group (MCAQ’s total score is in the bottom 27%). These findings suggest that all items in the questionnaire demonstrate good discrimination. Detailed results are presented in Table 2.

#### 2.4.2. EFA

The KMO measure of sampling adequacy was 0.932, and Bartlett’s test of sphericity was significant (χ^2^ = 5082.892, *df* = 45, *p* < 0.001), which meant that the data were suitable for EFA.

The EFA results led to the removal of Item 1 and Item 2, as they formed a factor with fewer than three items and had factor loadings below 0.4 on Factor 1. The final MCAQ thus consisted of 8 items. A single factor accounted for 74.01% of the total variance. The factor loadings and communalities for the initial MCAQ are presented in Table 3. Factor loadings and communalities for the MCAQ after removing items 1 and 2 are presented in Table 4.

#### 2.4.3. The MCAQ’s Reliability and Validity

The MCAQ’s Cronbach’ alpha was 0.957, which demonstrated that the MCAQ had good internal consistency. The CFA showed that the model fit the data well: χ^2^/df = 4.20, CFI = 0.969, TLI = 0.956, SRMR = 0.021, RMSEA = 0.079 [0.062, 0.097]. These results show that the MCAQ is reliable and has good structural validity.

The criterion validity analysis (see Table 5) revealed that MCAQ’s total score was significantly positively correlated with aversion to violence (this scale was developed by our team and has not yet been published), aversion to harm (Miller et al., 2014), and the anticipated guilt/shame (van Gelder et al., 2022). Conversely, there were weak negative correlations between moral cost and moral disengagement (Caprara et al., 2009), aggression (Buss & Perry, 1992), reactive-proactive aggression (Raine et al., 2006), and cyberbullying (Antoniadou et al., 2016).

## 3. Study 2

### 3.1. Participants

In Study 2, a priori power analysis was performed utilizing G*Power 3.1 (Faul et al., 2009) to determine a suitable sample size. A minimum sample size of 89 participants was necessary to achieve an adequate power (0.95) for the multiple regression analysis, which included four predictors (gender, age, moral cost, and POEA), assuming α of 0.05 and effect size *f*^2^ of 0.15.

Consistent with Study 1, a convenience sampling was employed. College students were recruited from four universities in China. 789 online questionnaires were distributed during class intervals. After applying the same data screening protocol as described in Study 1, 749 valid questionnaires were retained (330 females; *M*age = 18.96 years, *SD* = 0.74), yielding an effective response rate of 94.93%.

### 3.2. Measures

Moral Cost of Aggression Questionnaire (MCAQ)—The final version of the MCAQ, established in Study 1, was utilized. Participants were asked to rate the extent to which they would generally feel discomfort, guilt, and shame when engaging in aggression (e.g., hitting someone), as well as the extent to which they would experience the pain and distress felt by the victim. This scale employed in this study (Sample 2) demonstrated good internal consistency (Cronbach’s α = 0.930). CFA indicated a good model fit: χ^2^/*df* = 2.01, CFI = 0.998, TLI = 0.994, SRMR = 0.006, RMSEA = 0.037 [0.000, 0.089].

Aggression Outcome Expectancy Questionnaire (AOEQ; Wei & Xia, 2023)—The AOEQ’s positive expectancy subscale was used to measure POEA. The positive expectancy subscale contains 15 items. For example, what extent do you expect that hitting someone would lead to satisfying or effective outcomes (e.g., gain respect or money) for yourself? There is a 7-point scale for scoring answers (1 = Extremely low, 7 = Extremely high). A higher mean score on this questionnaire reflects higher POEA. The questionnaire has shown good reliability and validity among Chinese college students (Wei & Xia, 2023). In this study, the Cronbach’s α coefficient is 0.983, and the CFA produced acceptable fit indices (χ^2^/*df* = 4.06, CFI = 0.995, TLI = 0.985, SRMR = 0.003, RMSEA = 0.064 [0.023, 0.112]).

Buss–Perry Aggression Questionnaire (BPAQ; Buss & Perry, 1992)—Aggression was assessed using the BPAQ, which includes “I get into fights a little more than the average person.” BPAQ used a 5-point Likert scale, with “1 = Completely false for me” and “5 = Completely true for me.” A higher mean score means higher aggression. The Chinese version of the BPAQ has demonstrated adequate reliability and validity (Quan et al., 2019). The internal consistency coefficient for the scale in this study is 0.948, and the CFA showed good fit indices (χ^2^/*df* = 3.10, CFI = 0.998, TLI = 0.993, SRMR = 0.005, RMSEA = 0.053 [0.007, 0.103]).

### 3.3. Statistical Analysis

We used SPSS software (Version 26.0) to conduct common method bias tests, descriptive statistics, and correlation analyses on all research variables. Hayes PROCESS macro with 5000 bootstrap samples and 95% confidence intervals was used to estimate the mediation model (Hayes, 2017). In addition, considering that gender and age differences are known to exist in aggression (Lahey et al., 2000; Malonda-Vidal et al., 2021; Mancinelli et al., 2021), we tested the mediate model with gender and age as covariates.

### 3.4. Results

#### 3.4.1. Common Method Bias

Harman’s single-factor test (Podsakoff et al., 2003) showed that the first common factor explained 38.42% of the variance, which is below the critical threshold of 50%.

#### 3.4.2. Descriptive Statistics and Correlation Analysis

The results (Table 6) show that moral cost was negatively correlated with both POEA and aggression, and POEA was positively correlated with aggression. All correlations were below 0.70, indicating no multicollinearity issues.

#### 3.4.3. POEA as a Mediator

**Figure 1:**
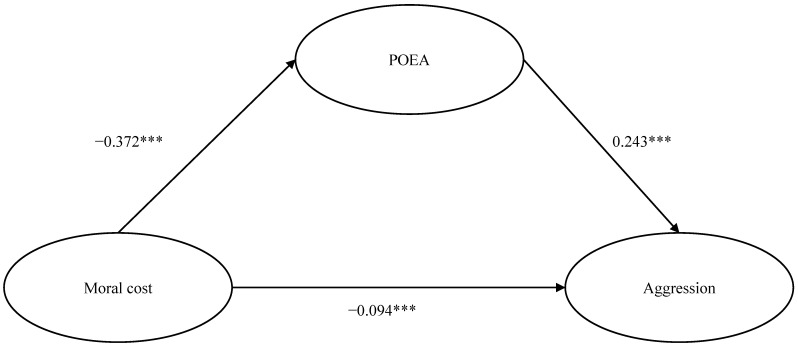
Mediation model for moral cost, POEA and aggression. Gender and age were omitted in the figure. *** *p* < 0.001.

We employed Model 4 from the PROCESS macro to examine the proposed mediation model, which posits POEA as a mediator in the relationship between moral cost and aggression. After controlling for sex and age, the results (see Figure 1) revealed that moral cost negatively predicted POEA (*β* = −0.372, *SE* = 0.03, *p* < 0.001) and aggression (*β* = −0.094, *SE* = 0.02, *p* < 0.001), and POEA positively predicted aggression (*β* = 0.243, *SE* = 0.02, *p* < 0.001). The indirect effect of moral cost on aggression through POEA was significant (*β* = −0.090, 95% CI = [−0.11, −0.07]). The indirect effect accounts for 48.99% of the total effect. This suggests that the mediation effect of POEA is significant, and that moral cost is indirectly associated with aggression through POEA. 

## 4. Discussion

Aggression is a significant concern among college students. Identifying moral protective factors against aggression and understanding their underlying mechanisms are key to creating effective prevention and intervention strategies. This study examined moral cost as a moral protective factor, exploring its role against aggression through the reduction in POEA. Firstly, we developed the MCAQ as a measurement instrument. Subsequently, the proposed mediation model involving moral cost, POEA, and aggression was tested on a college student sample. The findings indicated that moral cost was negatively associated with aggression and that this relationship was mediated by POEA.

### 4.1. The Moral Cost of Aggression Questionnaire (MCAQ)

This study developed the MCAQ, based on prior research (Abbink & Herrmann, 2011; Attanasi et al., 2019; Euler et al., 2017; Qu et al., 2022; Rothenhäusler et al., 2015; van Gelder et al., 2022), to provide a reliable instrument for examining the effect of moral cost on aggression. Notably, the MCAQ assesses the general dispositional tendency of moral cost. This represents a relatively stable personality trait, reflecting an individual’s baseline level of moral discomfort typically elicited by aggressive behavior. This distinguishes it from state moral cost, which is influenced by situational factors such as rationalization.

The results of item analysis and EFA showed that all items had good discrimination and clearly fit into a single-factor structure. Reliability and validity analysis showed that the MCAQ has excellent internal consistency and good structural validity. Furthermore, criterion validity analysis showed that there were significant negative associations between moral cost and various forms of aggression. However, the correlation coefficients were mostly in the weak range. This suggests that the relationship between moral cost and aggression may be moderated by other variables, such as moral disengagement. In summary, the MCAQ is a reliable, valid, and practical measurement tool, providing a solid foundation for testing the underlying mechanisms of aggression.

Compared to existing measurement tools, the unique contribution of the MCAQ is reflected in the following two aspects: First, in terms of the measured construct, the MCAQ aims to capture the comprehensive internal cost formed by integrating various moral emotions (such as guilt, shame, and harm aversion) and moral cognition when individuals engage in aggression, rather than separately assessing the cost arising from different components. This more closely aligns with the psychological process in which multiple moral emotions and moral cognition are weighed as an integrated cost in actual decision-making. Second, in terms of measurement method, the MCAQ requires participants to evaluate the moral cost for a series of specific aggressive behaviors (e.g., hitting someone, forcibly borrowing). This approach overcomes the limitation of existing tools that rely on general situational descriptions (e.g., “using violence”), making the assessment more closely aligned with real-world decision contexts and thereby significantly enhancing the ecological validity of the measurement.

### 4.2. The Mechanism of Moral Cost in Inhibiting Aggression: The Mediating Role of POEA

First, our results indicated that moral cost could predict aggression both directly and indirectly, supporting the hypothesis that moral cost is one of the moral protective factors against aggression. Second, mediation analysis revealed that the effect of moral cost on aggression was partially mediated by POEA. This result is consistent with previous research indicating that aggressive cognitions (e.g., moral disengagement) mediate the relationship between moral emotions and aggression (Cen et al., 2025; Ouvrein et al., 2018; Shen et al., 2023). Our results support our hypothesis that POEA mediates the relationship between moral cost and aggression. A key contribution of this study is the conceptualization of moral cost as a discount factor in an individual’s decision calculus, and the specification and empirical evaluation of a value-discounting mechanism in which moral cost is associated with lower aggression through its link to reduced subjective value of aggressive outcomes (i.e., lower POEA).

This moral discounting mechanism can be effectively understood within the cost–benefit analysis framework of behavioral decision-making (Becker, 1968). Within this framework, the “net utility” of any action equals its perceived benefit minus its perceived cost (Barile et al., 2025; Grasmick et al., 1993). The findings are consistent with a discounting model in which moral cost is associated with lower perceived net utility (i.e., subjective value/POEA) of aggression. Consequently, the inhibition effect of moral factors on aggression can be understood as a utility-based, computational process, providing new empirical support for the “rational” foundation of moral behavior.

It is important to emphasize that the mediation analysis in this study is based on cross-sectional data, and therefore cannot establish the causal relationship among the variables. Although the findings are consistent with the theoretical model (moral cost → POEA → aggression), alternative explanations (such as reverse causality) cannot be completely ruled out. We tested the alternative model (POEA → moral cost → aggression) and found its indirect effect is significant (*β* = 0.037, 95% CI = [0.02, 0.05]). This result suggests that future research should employ experimental design or longitudinal design to examine the causal relationship and underlying mechanisms between moral cost and POEA.

Furthermore, from the perspective of dual-process theories (Chaiken & Trope, 1999), and in particular sociological dual-process approaches such as situational action theory (Wikström, 2014) or the model of frame selection (Esser & Kroneberg, 2015), it is plausible for moral cost to function as a moderator: the higher the moral costs, the weaker the effect of POEA on aggression. To examine this possibility, we tested a moderation model in which moral cost moderates the relationship between POEA and aggression. The results showed that the interaction between moral cost and POEA was not significant (*β* = 0.002, *p* = 0.124), indicating that the strength of the association between POEA and aggression does not depend on the level of an individual’s moral cost. Nevertheless, future research should further explore the potential moderating role of moral cost in the relationship between POEA and aggression.

### 4.3. Contributions and Limitations

This research offers several contributions. First, it created a tool for measuring the trait moral costs of aggression. Secondly, this study suggests a new protective factor (e.g., moral cost) against aggression. Establishing moral cost as a protective factor against aggression enriches research on the relationship between morality and aggression. Third, this study examines a potential mechanism linking moral cost to aggression—namely, through its association with POEA. This finding offers a new perspective on how moral factors influence aggression. This finding moves beyond the general conclusion that morality inhibits aggression and uses the notion of “moral cost reduces subjective value” to specify its cognitive pathway of aggression. This provides an empirical bridge connecting moral psychology with behavioral decision theory. Fourth, the results provide guidance for the intervention of aggression. Interventions can aim not only to increase moral costs by fostering empathy and harm aversion but also to cultivate accurate perceptions of the outcomes of aggression.

This study has several limitations. First, we did not distinguish separate dimensions of moral cost. Instead, we integrated the moral discomfort elicited by both moral cognition and moral emotions into a single, comprehensive cost, thereby capturing the overall moral cost individuals anticipate when considering aggression. We also note that moral cost has the potential for a multidimensional structure in theory, and one of the important directions for future research is to explore the subdimensions of moral cost in order to further uncover the mechanisms of its different components. Second, this study developed and validated the MCAQ within the Chinese cultural context. We have not yet examined its cross-cultural applicability. Therefore, the current applicability of this questionnaire is limited to similar cultural and linguistic settings, and future research should further verify the suitability of the MCAQ across diverse cultures. Third, our sample consisted of college students; the findings’ applicability to other groups remains to be established. Fourth, the use of self-report measures may introduce social desirability bias. Thus, replicating our results using alternative methods (e.g., experimental designs) would be beneficial. Finally, moral cost was measured as a personality-related disposition rather than experimentally manipulated; as such, unmeasured confounders cannot be ruled out. Furthermore, the cross-sectional design precludes causal conclusions. Future research employing experimental or longitudinal designs is needed to strengthen causal inferences.

## 5. Conclusions

To elucidate the effects of moral protective factors (e.g., moral cost) on aggression and their underlying mechanisms, this study developed a moral cost questionnaire and tested the proposed mediation model among college students. The results are consistent with the hypothesis that moral cost is negatively associated with aggression and that this relationship is mediated by POEA. This research provides preliminary evidence for a pathway through which moral protective factors may be linked to aggression, offering potential implications for both theory and intervention development.

## Figures and Tables

**Table 1:** The Initial MCAQ’s Items.

Items	Please Imagine Yourself Engaging in the Following Behaviors and Rate the Extent to Which You Would Generally Feel Discomfort, Guilt, and Shame When Engaging in the Behavior, as Well as the Extent to Which You Would Experience the Pain and Distress Felt by the Victim.
1	Grabbing, pushing, or shoving someone
2	Arguing or quarreling
3	Sowing discord among others
4	Excluding or isolating a classmate (or classmates)
5	Hitting someone (e.g., punching, kicking, slapping)
6	Damaging or destroying someone else’s belongings
7	Swearing at or verbally insulting someone
8	Intentionally leaking or exposing someone’s private information
9	Forcibly borrowing, demanding, or snatching others’ possessions
10	Mocking or harassing others online through platforms such as QQ, WeChat, Weibo, or forums

**Table 2:** The Initial MCAQ’s Item Analysis Results (*N* = 516).

Items	*r*	*t*	Items	*r*	*t*
1	0.650 ***	16.791 ***	6	0.904 ***	28.384 ***
2	0.604 ***	14.436 ***	7	0.731 ***	19.249 ***
3	0.842 ***	26.356 ***	8	0.903 ***	30.702 ***
4	0.871 ***	27.717 ***	9	0.906 ***	29.723 ***
5	0.880 ***	29.371 ***	10	0.868 ***	29.835 ***

*r* item-total correlation, *t* critical ratio. *** *p* < 0.001.

**Table 3:** Factor Loadings and Communalities for the Initial MCAQ.

Items	Factor Loadings		Communalities
	Factor 1	Factor 2	
1	0.289	0.740	0.632
2	0.187	0.854	0.764
3	0.694	0.435	0.671
4	0.835	0.269	0.770
5	0.795	0.352	0.756
6	0.862	0.308	0.839
7	0.516	0.470	0.487
8	0.908	0.239	0.881
9	0.909	0.243	0.886
10	0.827	0.272	0.758

**Table 4:** Factor Loadings and Communalities for the MCAQ.

Items	Factor Loadings	Communalities
3	0.799	0.638
4	0.881	0.776
5	0.870	0.757
6	0.916	0.840
7	0.643	0.414
8	0.930	0.866
9	0.933	0.871
10	0.871	0.759

**Table 5:** Results of the Criterion Validity Analysis (*N* = 522).

Variables	*M* (*SD*)	1
1. moral cost	4.87 (1.52)	—
2. aversion to violence	5.10 (1.95)	0.480 ***
3. aversion to harm	4.75 (1.81)	0.440 ***
4. anticipated guilt/shame	4.85 (2.15)	0.439 ***
5. moral disengagement	1.73 (0.67)	−0.235 ***
6. aggression	1.84 (0.80)	−0.249 ***
7. reactive aggression	1.44 (0.38)	−0.238 ***
8. proactive aggression	1.13 (0.33)	−0.261 ***
9. cyber bullying	1.28 (0.68)	−0.166 ***

*M*, *mean*; *SD*, *standard deviation*. *** *p* < 0.001.

**Table 6:** Means, standard deviations, and correlations of the research variables.

	*M*	*SD*	1	2
1. moral cost	5.11	1.23		
2. POEA	1.95	1.18	−0.396 ***	
3. aggression	2.07	0.66	−0.359 ***	0.510 ***

*M*, *mean*; *SD*, *standard deviation*. *** *p* < 0.001.

## Data Availability

The data and code that support the findings of this study are available from Open Science Framework (OSF; https://osf.io/j4ny9/overview?view_only=b8fce120b1a94b4bb7fcd019890780d7, accessed on 9 December 2025).

## Abbreviations

The following abbreviations are used in this manuscript:

MCAQ	Moral Cost of Aggression Questionnaire
POEA	Positive Outcome Expectancies for Aggression
